# Phospholipid profiles and hepatocellular carcinoma risk and prognosis in cirrhotic patients

**DOI:** 10.18632/oncotarget.26738

**Published:** 2019-03-15

**Authors:** Alexia Karen Cotte, Vanessa Cottet, Virginie Aires, Thomas Mouillot, Maud Rizk, Sandrine Vinault, Christine Binquet, Jean-Paul Pais de Barros, Patrick Hillon, Dominique Delmas

**Affiliations:** ^1^ University of Bourgogne, Franche-Comté, Dijon, France; ^2^ INSERM U1231 “Lipids, Nutrition, Cancer”, Research Team Cancer and Adaptive Immune Response (CADIR), Dijon, France; ^3^ INSERM U1231 “Lipids, Nutrition, Cancer”, Research Team Epidemiology and Clinical Research in Digestive Oncology (EPICAD), Dijon, France; ^4^ Department of Hepatogastroenterology, University Hospital, Dijon, France; ^5^ Lipidomic Platform, Dijon, France; ^6^ Inserm, Clinical Investigation Center, Dijon, France

**Keywords:** hepatocellular carcinoma, cirrhosis, phospholipids, biomarker, case–control study

## Abstract

**Background:**

Hepatocellular carcinoma (HCC) is the fifth most common cancer worldwide. Phospholipids are now well-recognised players in tumour progression. Their metabolic tissue alterations can be associated with plasmatic modifications. The aim of this study was to evaluate the potential of the plasma phospholipid profile as a risk and prognostic biomarker in HCC.

**Methods:**

Ninety cirrhotic patients with (cases) or without HCC (controls) were studied after matching for inclusion centre, age, gender, virus infection, cirrhosis duration and Child-Pugh grade. High-performance liquid chromatography coupled with tandem-mass spectrometry was used to quantify the main species of seven categories of phospholipids in plasma.

**Results:**

Elevated concentrations of phosphatidylcholine (PC) 16:0/16:1 (p=0.0180), PC 16:0/16:0 (p=0.0327), PC 16:0/18:1 (p=0.0264) and sphingomyelin (SM) 18:2/24:1 (p=0.0379) and low concentrations of lysophosphatidylcholine 20:4 (0.0093) and plasmalogen-phosphatidylethanolamine (pPE) 16:0/20:4 (p=0.0463), pPE 18:0/20:4 (p=0.0077), pPE 18:0/20:5 (p=0.0163), pPE 18:0/20:3 (p=0.0463) discriminated HCC patients from cirrhotic controls. Two ceramide species were associated with increased HCC risk of death while lysophospholipids, a polyunsaturated phosphatidylinositol, some PC and SM species were associated with low risk of death in HCC patients in 1 and/or 3 years.

**Conclusion:**

This study identified phospholipid profiles related to HCC risk in liver cirrhotic patients and showed for the first time the potential of some phospholipids in predicting HCC patient mortality.

## INTRODUCTION

Hepatocellular carcinoma (HCC) is the most common primary liver malignancy worldwide. Patients with early detection of HCC have a relatively good prognosis, with a 5-year survival rate of more than 70%. Most patients with hepatocellular carcinoma are diagnosed with late-stage disease resulting in an overall 5-year survival rate of less than 20%. Its aggressiveness explains its second rank in cancer-related death [1]. In Western countries, HCC occurs predominantly in viral, alcoholic, hemochromatosis or metabolic cirrhosis [2]. Most HCC cases are diagnosed at an advanced stage due to late diagnosis limiting treatment possibilities. Consequently, better knowledge of individual risk factors for HCC in persons with cirrhosis is the basis for disease management and for designing preventive or treatment strategies. The current issue is to provide markers making it possible to identify, among cirrhotic patients, HCC high-risk individuals who may benefit from intensive screening. Therefore, numerous studies have focused on finding new serologic or plasmatic biomarkers to combine them or not with existing diagnostic tests [3]. Among the new markers studied, lipids and particularly phospholipids retain the scientific community’s attention. Alteration of tumour metabolism including lipid metabolism appears to be involved in carcinogenesis. This alteration of lipid metabolism is marked by an increase in *de novo* synthesis of all lipid classes. Two key steps in lipid synthesis that have been thoroughly described in carcinogenesis development are catalyzed by ATP citrate lyase (ACLY) and fatty acid synthase (FASN), where their inhibition could induce apoptosis and reduce tumour growth of cancer cells [4, 5]. More recently, phospholipids appear to be involved in tumour progression, through their structural function in membrane composition and their cell-signalling functions [6, 7]. In HCC, a metabolomic analysis revealed that phosphatidylcholine (PC) could be the predominant species in HCC tissue associated with overexpression of the lysophosphatidyl-choline acyltransferase 1 (LPCAT1) involved in the re-acylation of lysophosphatidylcholine (LPC) into PC [8]. In same manner, we have recently demonstrated that a lysophosphatidylcholine acyltransferase isoform, LPCAT2, and PC are also involved in chemoresistance in colorectal cancer [9]. Cancer development associated with phospholipid metabolism alterations also affect circulating phospholipids. Indeed, a few interventional studies in humans suggest that the plasma level of phospholipids (PLs) could serve as predictive biomarkers of progression of various cancers, such as lysophosphatidic acid (LPA) for gynaecological cancers [10, 11], lysophosphatidylcholine (LPC) for ovarian cancers [12], colorectal cancers [13] and prostate cancer where ether-phosphatidylcholine (e PC) 38:5, PC 40:3, PC 42:4 serum levels are increased [14]. This modulation of circulating phospholipid levels was also found in the serum of hepatitis B-related HCC for which Passos-Castilho et al. showed a general decrease in phosphatidylserine (PS), phosphatidylinositol (PI) and PC [15]. Patterson and colleagues showed that cirrhotic patients present higher plasma LPC concentrations compared to HCC patients [16]. Nevertheless, this study was followed by a metabolomic analysis based on the serum of hepatitis B-related cirrhotic patients and HCC patients showing an increase in two LPC species and five PC species in HCC patients [17]. As highlighted by Patterson, numerous metabolomic studies are focused on Chinese populations. Furthermore, despite the increasing number of studies focusing on the relation between phospholipids and HCC, the small number of patients, the heterogeneity of the results and the fewer matching parameters for case–control studies led us to examine the potentiality of phospholipids to diagnose and predict the progression of HCC in cirrhotic liver, an important challenge to better knowledge of the individual risk factors for HCC.

In this paper, according to the literature [18], we studied the concentration of the major classes of circulating complex lipids of 90 well-described European patients with cirrhosis – 45 cirrhotic patients without HCC (controls) and 45 cirrhotic patients with HCC (cases) – randomised in the CiRCE study (Cirrhose et Risque de Cancer dans le Grand-Est) after matching for age, gender, virus infection, duration of cirrhosis, Child-Pugh score and inclusion centre. These matching parameters were selected considering their potential relationship to HCC incidence and/or lipid modulations [19, 20]. High-performance liquid chromatography coupled with tandem-mass spectrometry (HPLC-MS/MS) revealed for the first time on a European cohort that only some phospholipid species were elevated (phosphatidylcholine, sphingomyelin and plasmalogen-phosphatidylethanolamine), thus discriminating HCC patients from their cirrhotic controls. More importantly, we highlight that two ceramide species were associated with increased risk of death from HCC.

## RESULTS

### Patient characteristics

The median age of controls and cases was 59.9 (range, 55.7–69.4) and 62.4 (range, 58.2–71.6) years, respectively; the global sex ratio was eight males for one female in controls and cases [19, 20]. The main etiologies of cirrhosis were alcohol (51.1 and 64.4% in cases and controls, respectively, followed by hepatitis C virus (31.1 and 20.0%, respectively). In most patients liver function was normal with the Child-Pugh score between A5 and B7 (Table 1). Case patients mainly presented nodular HCC (multinodular: 51.1%, uninodular: 35.6%). No significant differences in daily alcohol consumption, smoking status, BMI, diabetes, statin and other hypolipemic treatment were observed between cases and controls (Table 1). Concerning biological characteristics, total cholesterol, LDL (low-density lipoproteins), HDL (high-density lipoproteins) and triglycerides were not statistically different between cases and controls. Gamma-glutamyl transferase (GGT) (p=0.0139) and alpha-foetoprotein (AFP) (p<0.0001) values were higher in cases than in controls (Table 2).

**Table 1:** Demographic and clinical characteristics of the 90 cirrhotic patients with (cases) and without (controls) HCC

	Controls n=45	Cases n=45	P-value
**Matching criteria (+ centre)**			
**Age (years), median [IQR]**	59.9 [55.7–69.4]	62.4 [58.2–71.6]	-
**Gender, n (%) :**			
**Male**	40 (88.9)	40 (88.9)	-
**Female**	5 (11.1)	5 (11.1)	
**Virus infection, n (%)**	16 (35.6)	16 (35.6)	-
**Time between cirrhosis diagnosis and inclusion in the study (years), median [IQR]**	2 [1–6]	2 [0–5]	-
**Child-Pugh score, n (%):**			
**A5 to B7**	34 (75.6)	34 (75.6)	-
**B9 to C13**	11 (24.4)	11 (24.4)	
**Clinical characteristics**			
**Etiology of cirrhosis, n (%):**			
**Alcohol**	23 (51.1)	29 (64.4)	
**B virus**	1 (2.2)	7 (15.6)	
**C virus**	14 (31.1)	9 (20.0)	
**Others**	7 (15.6)	0	
**Tumour characteristics, n (%):**			
**No tumour**	45	-	-
**Uninodular**	-	16 (35.6)	
**Multinodular**	-	23 (51.1)	
**Diffuse and/or metastases**	-	6 (13.3)	
**Excessive alcohol consumption, n (%):**			
**Never**	8 (17.8)	9 (20.0)	0.8674
**Former**	17 (37.8)	20 (44.4)	
**Current**	20 (44.4)	16 (35.6)	
**Smoking status, n (%):**			
**Never**	10 (22.2)	14 (31.1)	0.2872
**Former**	23 (51.1)	18 (40.0)	
**Current**	12 (26.7)	13 (28.9)	
**BMI ≥ 25 kg/m^2^, n (%)**	27 (61.4)	30 (66.7)	0.6776
**Diabetes, n (%)**	21 (46.7)	17 (37.8)	0.5235
**Treatments, n (%):**			
**Hypolipemic**	11 (24.4)	9 (20.0)	0.7905

**Table 2:** Biological characteristics of the 90 cirrhotic patients with (cases) and without (controls) HCC

Biology, median [IQR]	Controls n=45	Cases n=45	P-value
**Total cholesterol (g/L) [25 MD]**	3.79 [3.21–5.11]	4.21 [3.11–4.85]	0.4777
**LDL (g/L) [25 MD]**	2.07 [1.73–2.91]	2.30 [1.76–3.00]	0.3732
**HDL (g/L) [25 MD]**	1.11 [0.77–1.48]	1.11 [0.48–1.69]	0.7911
**Triglycerides (g/L) [25 MD]**	1.09 [0.91–1.37]	1.01 [0.88–1.47]	0.1480
**Anti-diabetic prescription, n (%)**	16 (35.6)	14 (31.1)	
**Glycaemia (μmol/L)**			
Treated group [10 MD]	7.0 [6.0–10.1]	6.7 [5.7–7.4]	-
Non-treated group [15 MD]	5.1 [4.4–5.7]	5.0 [4.4–5.7]	-
**Insulinaemia (μIU/mL)**			
Treated group [20 MD]	16.9 [7.3–20.7]	23.3 [13.3–26.8]	-
Non-treated group [36 MD]	9.8 [5.0–19.8]	11.5 [7.9–16.4]	-
**AFP (IU/mL) [1 MD]**	4 [3–7]	23 [6–157]	< 0.0001
**Transaminases (IU/L) [1 MD]**	37 [25–72]	41 [29–55]	0.8814
**GGT (IU/L) [2 MD]**	147 [66–240]	205 [120–298]	0.0139
**Bilirubin (μmol/L) [2 MD]**	18 [12–31]	22 [14–34]	0.2107

Abbreviations: LDL, low-density lipoprotein; HDL, high-density lipoprotein; AFP, alpha foetoprotein; GGT, gamma-glutamyl transferase; MD, missing data.

### Distinctive plasma lipid profiles of liver cirrhosis with and without HCC

Circulating phospholipid profiles have been previously described as modified in different manners in various cancers with some contradictory results in HCC cases [16]. To evaluate phospholipid levels in our cohort, we analysed the plasma of the 90 cirrhotic patients using the HPLC-MS/MS approach with multiple reaction monitoring (MRM). We first made qualitative analyses to identify each lipid species from the HPLC spectrum according to carbon and unsaturation numbers. Then we quantified each species using the internal standard. At first this showed no difference in the concentration of total plasma phospholipids and biliary acid concentration between cases and controls (data not shown). Nevertheless, in a second step, a comparison of each phospholipid species in each lipid category highlighted that only PC 16:0/16:1 (p=0.018), PC 16:0/16:0 (p=0.033), PC 16:0/18:1 (p=0.026) and SM 18:2/24:1 (p=0.038) concentrations were increased in cases compared to controls.

On the other hand, analyzing lysophospholipid species, which are formed by hydrolysis of phospholipids, we observed that only the LPC 20:4 (p=0.009) concentration was lower in cases than in controls (Table 3 and Supplementary Table 2).

**Table 3:** Discriminating phospholipids between HCC cases and matched controls

Phospholipids^*^	Min	Max	All n=90	Controls n=45	Cases n=45	P-value
**PC 16:0/16:1**	4.27	113.8	27.7 [17.37–38.42]	25.39 [16.01–35.21]	29.96 [22.45–40.68]	0.018
**PC 16:0/16:0**	5.22	61.82	21.84 [18.38–29.01]	20.85 [16.9–24.92]	23.02 [19.77–29.5]	0.0327
**PC 16:0/18:1**	49.51	395.82	207.38 [186.29–240.55]	198.79 [166.19–229]	225.95 [194.24–250.62]	0.0264
**LPC 20:4**	1.34	12.96	4.02 [3.03–5.14]	4.3 [3.48–6.05]	3.66 [2.89–4.53]	0.0093
**pPE 16:0/20:4**	0.05	0.99	0.37 [0.29–0.49]	0.42 [0.3–0.55]	0.35 [0.28–0.43]	0.0463
**pPE 18:0/20:4**	0.04	2.03	0.75 [0.55–1.03]	0.89 [0.62–1.17]	0.63 [0.53–0.85]	0.0077
**pPE 18:0/20:5**	0	0.21	0.07 [0.06–0.1]	0.09 [0.06–0.11]	0.06 [0.05–0.09]	0.0163
**pPE 18:0/20:3**	0.01	0.31	0.1 [0.07–0.14]	0.12 [0.08–0.15]	0.09 [0.06–0.13]	0.0463
**SM d18:2/24:1**	6.46	38.46	22.39 [18.96–26.76]	20.81 [18.49–25.78]	23.45 [20.68–28.8]	0.0379

* Values are median [IQR] of μmol/L of plasma.

In a similar manner, among ether-phospholipids, plasmalogen-phosphatidylethanolamine (pPE) 16:0/20:4 (p=0.046), pPE 18:0/20:4 (p=0.008), pPE 18:0/20:5 (p=0.016), pPE 18:0/20:3 (p=0.046) concentrations were also lower in cases than in controls (Table 3 and Supplementary Table 2).

**Figure 1:**
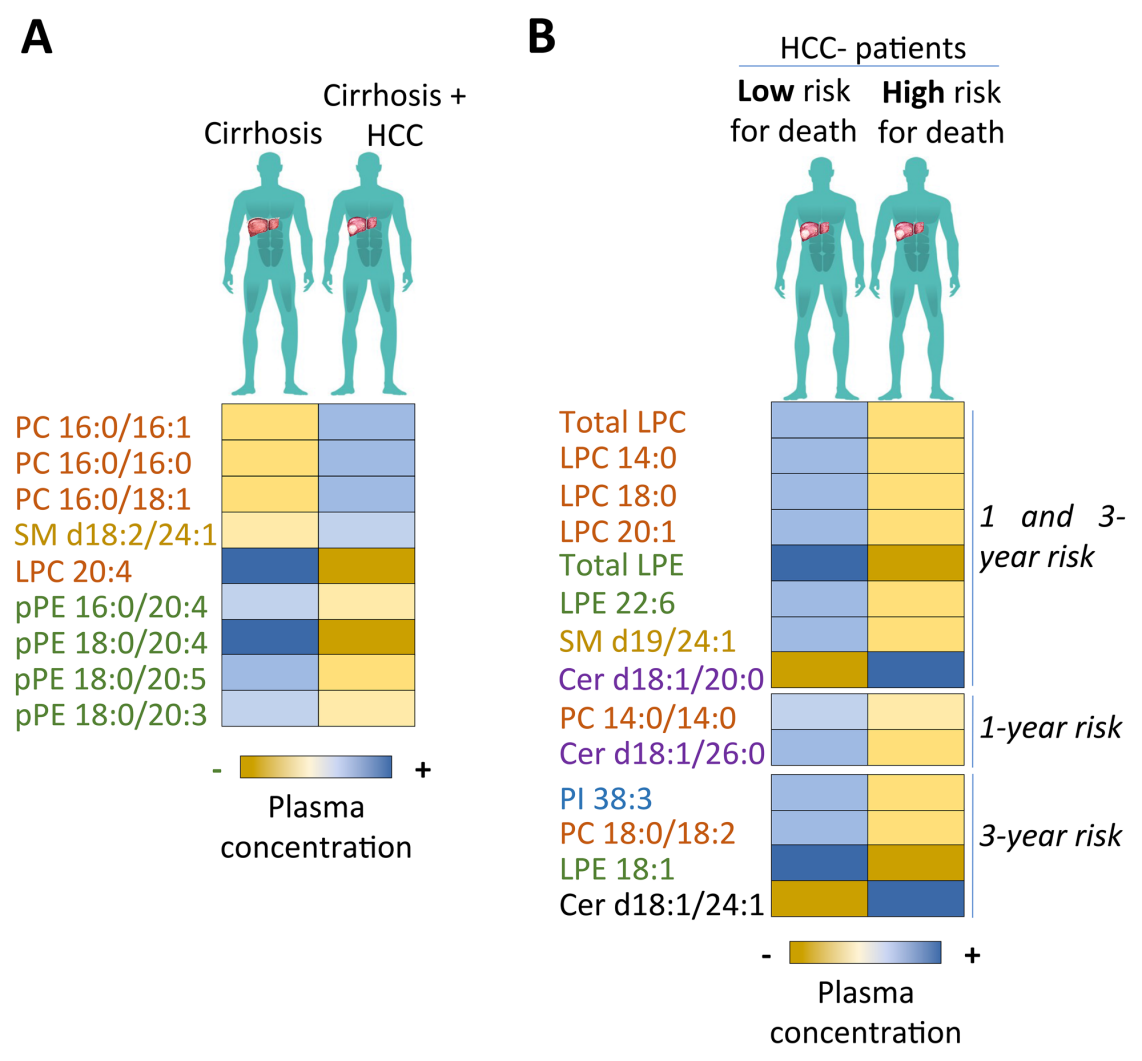
Phospholipid profiles in cirrhotic patients depending on HCC presence and mortality risk **(A)** Phospholipid profiles according to plasma concentration of each species in cirrhotic patients with or without HCC. Blue rectangles symbolise a higher concentration of the corresponding lipid in comparison to control patients. Yellow rectangles symbolise a lower concentration of the corresponding lipid in comparison to control patients. Only phospholipids with statistical differences are represented. **(B)** Phospholipid profiles according to the plasma concentration of each species in HCC patients with a low or a high risk of mortality at 1 year or 3 years after their inclusion in the study. Blue rectangles symbolise a higher concentration of the corresponding lipid in comparison to the median value of this lipid in the HCC patient group. Yellow rectangles symbolise a lower concentration of the corresponding lipid in comparison to the median value of this lipid in the HCC patient group. Only phospholipids with statistical differences at 1 year or 3 years or both are represented.

Altogether, these data confirm an alteration of the qualitative and quantitative phospholipid profile in cirrhotic patients with HCC *versus* cirrhotic patients without HCC with a specificity of the levels of certain circulating species (Figure 1A).

### Relation between phospholipid profile and HCC patient survival

In accordance with previous results, we evaluated the prognostic potential of all phospholipid species based on patient follow-up. Patients were followed up once a year for 5 years after their inclusion in the clinical study. We analysed the probability of death 1 and 3 years after the beginning of inclusion for HCC patients who had a quantity above the median for each lipid species. Table 4 summarises the univariate and multivariate analysis of 1- and 3-year mortality in HCC patients according to the median split for each phospholipid species. Phospholipid species were included in multivariate analysis when there was a trend in univariate analysis with p<0.10. In multivariate analysis, several phospholipids were associated with a low mortality rate in HCC patients at 1 and 3 years: total LPC (1 year: p=0.042; 3 years: p=0.022); LPC 14:0 (1 year: p=0.024; 3 years: p=0.015); LPC 18:0 (1 year: p=0.049; 3 years: p=0.016); LPC 20:1 (1 year: p=0.008; 3 years: p=0.033); total LPE (1 year: p=0.017; 3 years: p=0.008); LPE 22:6 (1 year: p=0.030; 3 years: p=0.001); SM d19:0/24:1 (1 year: p=0.010; 3 years: p=0.020). PC 14:0/14:0 was only associated with a low 1-year death rate (p=0.038) while PC 18:0/18:2 (p=0.015); LPE 18:1 (p=0.008); PI 38:3 (p=0.028) were only associated with a low 3-year mortality rate (Table 4 and Figure 1B).

**Table 4:** Proportions of phospholipid species and death rates of cirrhotic patients with HCC in 1 and 3 years

	Univariate analysis				Multivariate analysis			
	1-year mortality		3-year mortality		1-year mortality		3-year mortality	
Parameter	HR [CI]	P-value	HR [CI]	P-value	HR [CI]	P-value	HR [CI]	P-value
**PC 14:0/14:0**	0.393 [0.157–0.987]	0.047	-	-	0.331 [0.117–0.940]	0.038	-	-
**PC 18:0/18:2**	-	-	0.507 [0.249–1.030]	0.061	-	-	0.318 [0.127–0.797]	0.015
**LPC**	0.361 [0.144–0.908]	0.030	0.556 [0.277–1.115]	0.098	0.300 [0.094–0.957]	0.042	0.313 [0.116–0.845]	0.022
**LPC 14:0**	0.363 [0.145–0.915]	0.032	0.562 [0.280–1.128]	0.105	0.247 [0.074–0.829]	0.024	0.367 [0.164–0.820]	0.015
**LPC 18:0**	0.361 [0.144–0.908]	0.030	0.468 [0.232–0.944]	0.034	0.314 [0.099–0.993]	0.049	0.299 [0.112–0.801]	0.016
**LPC 20:1**	0.392 [0.156–0.983]	0.046	0.557 [0.277–1.120]	0.101	0.251 [0.091–0.696]	0.008	0.434 [0.202–0.933]	0.033
**LPE**	0.361 [0.143–0.907]	0.030	0.489 [0.243–0.987]	0.046	0.245 [0.077–0.778]	0.017	0.301 [0.124–0.728]	0.008
**LPE 18:1**	-	-	0.482 [0.239–0.972]	0.041	-	-	0.301 [0.124–0.732]	0.008
**LPE 22:6**	0.466 [0.190–1.142]	0.095	0.485 [0.240–0.978]	0.043	0.256 [0.077–0.847]	0.03	0.189 [0.070–0.512]	0.001
**PI 38:3**	-	-	0.539 [0.267–1.086]	0.084	-	-	0.304 [0.105–0.881]	0.028
**SM d19:0/24:1**	04.68 [0.091–1.148]	0.097	0.452 [0.223–0.919]	0.028	0.195 [0.056–0.680]	0.010	0.243 [0.073–0.803]	0.020
**Cer d18:1/20:0**	1.787 [0.729–4.378]	0.204	1.206 [0.602–2.415]	0.597	3.227 [1.033–10.084]	0.044	5.595 [2.101–14.162]	0.003
**Cer d18:1/24:1**	-	-	1.231 [0.615–2.467]	0.557	-	-	5.454 [2.101–14.162]	0.000
**Cer d18:1/26:0**	0.521 [0.213–1.277]	0.154	-	-	0.282 [0.091–0.874]	0.028	-	-

Death within 1 year, 20/45 (44.4%); death within 3 years, 32/45 (71.1%)

Variables of adjustment for multivariate analysis: Child-Pugh; treatment; centre; diabetes.

In the second step, we decided to include ceramides in multivariate analysis despite the absence of a trend in univariate analysis (p<0.600). Ceramides have already been described in detail in terms of their anti-tumour function. Nevertheless, several studies started to describe their functional duality in organ injury promoting the progression of pathologies [21, 22]. In the present study, we highlighted that Cer d18:1/20:0 (1 year: p=0.044; 3 years: p=0.003) was associated with both high 1-year and 3-year mortality rates while Cer d18:1/24:1 (p=0.000) was only linked with elevated 3-year mortality. Nevertheless, Cer d18:1/26:0 (p=0.028) was associated with a low risk of death at 1 year in HCC patients (Table 4 and Figure 1B).

Collectively these data highlight that determining a specific phospholipid and ceramide species profile is necessary to predict HCC risk in liver cirrhotic patients and that some phospholipids could potentially be used to predict HCC patient mortality.

## DISCUSSION

Based on a non-invasive targeted lipidomic method using HPLC-MS/MS, for the first time we have highlighted potential plasma phospholipid biomarkers of HCC risk in 90 cirrhotic patients with or without HCC in a European cohort from the CiRCE study. For this case–control study, we previously demonstrated a relationship between PNPLA3 (patatin-like phospholipase domain-containing protein 3) GG status and oxidative DNA damage, which may explain the excessive risk of cancer in patients with cirrhosis [23]. According to the literature [18] and considering that some specific phospholipid species could be associated with the progression of HCC characterised by high inflammation and oxidative status [24], we investigated the main species of eight phospholipid classes: PC, LPC, phosphatidylethanolamine (PE), lysophosphatidylethanolamine (LPE), sphingomyelin (SM), ceramide (Cer), dihydroceramide (DHcer) and PI. A matched case–control study was conducted to free our analysis of possible bias stemming from risk factors known for modulating HCC risk and/or lipid metabolism such as virus infection [20]. We found nine phospholipids discriminating presence or absence of HCC in cirrhotic patients and 14 phospholipids differentially associated with risk of death in HCC patients. Early detection of cancer and identification of new HCC prognostic factors are important to improving patient management. Phospholipidomic analysis is a promising approach in biomarker research based on alteration in membrane composition and cell signaling in HCC.

Elevated levels of saturated and monounsaturated phospholipid species have already been observed in HCC tissues. A recent study highlighted lipid alteration of HCC tissues characterised by a decrease in PE, PS, PI and ceramide compared to non-tumoural liver. PC and LPC amounts were not changed. Saturated species were increased while polyunsaturated species of these phospholipid categories were reduced [25]. A study conducted by Morita et al. using MALDI-IMS (matrix-assisted laser desorption/ionisation-imaging mass spectrometry) revealed that PC 16:0/16:1 and PC 16:0/18:1 were significantly increased in tumours compared to non-tumour liver. These results were associated with an overexpression of LPCAT1 in tumour regions explained by the capacity of this enzyme to re-acylate specific LPC species into PC [8]. These results showing elevated PC species obtained in plasma of HCC and cirrhotic patients complete these earlier observations. Numerous papers have also reported dysregulation of PC synthesis enzymes in HCC such as choline kinase alpha (CKα) [26]. Moreover, among PC synthesis enzymes, phosphatidylethanolamine N-methyltransferase (PEMT) seems clearly downregulated during HCC [27], possibly explained by the fact that PEMT preferentially produces long-chain polyunsaturated PC [28]. A specific decrease of these species was already reported in HCC tissues [29] due to their anti-inflammatory functions [30]. In the present study PC 16:0/22:6 and 18:0/22:6 tended to decrease in patients with HCC. LPC 20:4 downregulation also partly confirms the results obtained by Patterson et al. [16], who pointed out a decrease in LPC 14:0, LPC 18:1 and LPC 20:4 using UPLC/QTOF-MS (ultra-performance liquid chromatography / quadrupole time-of-flight mass spectrometry) in HCC patients compared to non-matched cirrhotic patients. The high level of PC can be related to low levels of LPC, since it could result in their transformation by the action of phospholipase A2 to restore PC. A diminished level of LPC is often associated with inflammation status [31]. We also observed for the first time a significant decrease in four polyunsaturated plasmalogen PEs in the plasma of HCC patients compared to the cirrhotic control group. Plasmalogens are glycerophospholipids of cell membranes containing the vinyl-ether bond at the *sn*-1 position. Their levels are particularly low in the liver due to their predominant synthesis in hepatocytes and secretion in lipoproteins [32]. They act as serum antioxidants and specifically prevent oxidation of lipoproteins [33]. These results could indicate an increase in oxidative status during HCC development, which could also be involved in the activation of inflammation pathways [34]. These observations were also confirmed by the Cox regression models. Indeed, plasmatic high concentrations of several LPC species, polyunsaturated PI, and LPE were associated with a decreased risk of death in HCC patients, thus improving the prognosis. The disruption of phospholipid species equilibrium could be associated with the progression of HCC characterised by strong inflammation and oxidative status as we previously demonstrated in this cohort [23].

The literature amply describes the involvement of ceramide in the induction of apoptosis. In case of cancer, the increase in specific ceramides could be associated with the chemotherapy response [35]. Moreover, HCC and cirrhosis can induce extensive injuries to hepatocytes, which could be reflected by hepatocyte overproduction of ceramides with ongoing apoptosis [22], while PC 14:0/14:0, PC 18:0/18:2 and SM d19:0/24:1 plasma concentrations could reflect normal hepatocyte functions. Wang and colleagues reported that cirrhotic patients and HCC patients presented lower serum concentrations of PC species compared to healthy subjects. This observation argues that liver pathology decreases hepatocyte functions [17]. Several studies have also reported an alteration of lipid metabolism by hepatitis B and C viruses manifesting as a decrease in plasma triglycerides, cholesterol and LDL. These changes were also observed in patients with HCC [36, 37]. In fact, we observed no difference in total cholesterol, HDL, LDL and triglyceride amounts between matched cases and controls. These results suggest a general impact of cirrhosis on phospholipid content, which is also manifested in the present study by the absence of changes in the amount of total phospholipids, biliary acids, PI, PE, LPE, ceramides and DHceramides [38]. Moreover, it is intriguing that the low number and unusual species in plasma such as PC 14:0/14:0 and SM d19:0/24:1 could be easily associated with a slower progression of the disease, reinforced by the fact that saturated and monounsaturated phospholipids are often associated with cancer progression [18]. The same could be said for PC 18:0/18:2 containing 18:2 fatty acid, which is an n-6 fatty-acid mainly found in the diet. Also, it was surprising to find only one elevated SM species which had a differential level in patients with HCC corresp: SM d18:2/24:1. The variations of SM in plasma, serum or tissues differ depending on cancer types and studies [18]. SM is the major sphingolipid in blood and is associated with lipoproteins. Consequently, it is sometimes delicate to associate plasma phospholipid variation with tumour phenotype. These discrimination results could be due to an enrichment of PC 18:0/18:2 and SM d18:2/24:1 by a specific diet.

Several limitations of this study need to be mentioned. The number of patients was too small to conduct a complete study leading to pPE analysis in a Cox regression model and a real characterisation of biomarkers. These results need to be confirmed by including a greater number of patient levels. To conclude, our matched case–control study provides the opportunity to clarify the literature on phospholipids’ potency in discriminating cirrhosis and HCC using a targeted HPLC/MS/MS approach. The plasma concentration variation of nine phospholipids may be useful to identify HCC risk among cirrhotic patients. Interestingly, we show the possibility of using LPC, LPE and specific ceramide species concentrations as early predictive markers of HCC prognosis.

## MATERIALS AND METHODS

### Study design and population

This exploratory investigation is an ancillary study of CiRCE (Cirrhose et Risque de Cancer dans le Grand-Est), a multicentre case–control study analyzing environmental (alcohol, tobacco, viruses), nutritional and metabolic factors involved in liver carcinogenesis among cirrhotic patients. The 90 patients studied were randomised among the 1185 CiRCE cirrhotic patients treated in the university hospitals of the French Cancéropole du Grand Est (Besancon, Dijon, Metz, Nancy, Reims, Strasbourg) who were included from November 2008 through 2012. The study was approved by the Comité de Protection des Personnes Est (agreement no. 2008/09) and by the Agence Française de Sécurité Sanitaire des Produits de Santé (agreement no. 2008-A00023–52). All patients gave written informed consent to participate in this study. They were followed up for 5 years beginning at their inclusion. All clinical data were collected by the medical staff with the help of research assistants.

### Inclusion criteria

The diagnosis of cirrhosis, irrespective of its etiology, was based on histological confirmation by liver biopsy or, in the absence of biopsy, by a combination of clinical and biological signs of hepatocellular failure or portal hypertension and/or endoscopic features of portal hypertension and/or imaging characteristics of cirrhosis. All cases of HCC evolving in a context of cirrhotic liver were included. Criteria for the diagnosis of HCC were those defined by the European Association for the Study of the Liver. The absence of HCC in controls with liver cirrhosis at inclusion was assessed through high-quality imaging examinations (abdominal ultrasonography and/or computed tomography and/or magnetic resonance imaging) and AFP below 100 ng/mL during the 2 months preceding inclusion. Alcoholic cirrhosis was defined as alcohol consumption >14 units/week for females and >28 units/week for males or participant. The follow-up included hepatic ultrasonography and dosage of AFP every 6 months.

### Exclusion criteria

Patients under 35 years of age with progressive extrahepatic cancer, human immunodeficiency virus infection, acute alcoholic hepatitis, major somatic or psychiatric illness not compatible with inclusion in the study, or with non-HCC primary liver cancer were excluded.

### Samples

Plasma samples were collected, regardless of the cause of liver disease. Immediately after the blood test, plasma was extracted, frozen and stored at −80°C. Plasma glucose, HDL cholesterol, LDL cholesterol, triglycerides and liver enzymes were analysed using standard procedures. For this study, 45 cirrhotic patients without HCC (controls) and 45 cirrhotic patients with HCC (cases) were matched on inclusion university hospital, age, gender, presence of virus, the time between cirrhosis diagnosis and inclusion in the study, and the Child-Pugh score.

### Lipidomic analysis

#### Extraction

All extraction procedures were performed in 10-mL glass disposable centrifuge tubes. Plasma (150 μL) was diluted with saline solution; 50 μL of internal standard solution (Supplementary Table 1) prepared with a mix of chloroform (CHCl_3_)/methanol (MeOH)/water (H_2_O) (60/30/4.5) was added to each sample, followed by 1.5 mL of MeOH. Samples were shaken for 2 min before the addition of 5 mL Methyl tert-butyl ether (MTBE). Then the samples were shaken for 1 h followed by the addition of 1.25 mL of H_2_O. The samples were shaken for 10 min and centrifuged (10 000 *g* for 5 min, at 4°C). The organic phases were transferred to new glass tubes. The older tubes were washed with 2 mL of MTBE, shaken and centrifuged. The organic phases were transferred to the first new tubes. The solvent was partially evaporated at room temperature with a Speedvac apparatus for 25 min before addition of 200 μL MeOH and a new evaporation step for 1.5 h. Dried lipids were suspended in 200 μL of CHCl_3_/MeOH/H_2_O (60/30/4.5). The tubes were shaken for 2 min and centrifuged (10 000 *g* for 5 min at 4°C). The supernatant was transferred to a vial with a 200-μL reducer for MS analyses for LPE, PE, pPE; ceramide, dihydroceramide and PI; 2 μL of this vial were transferred to a new vial and mixed with 98 μL of CHCl_3_/MeOH/H_2_O (60/30/4.5) for PC, LPC and SM MS analyses.

#### LC/MS^2^

The samples were delivered into the electrospray ionisation (ESI)-MS ion source through a 1200 HPLC (Agilent Technologies) with an autosampler using a binary gradient of solvent A and B (Supplementary Table 1). Electrospray ionisation mass spectrometry (ESI-MS) was performed on a QQQ 6460 mass spectrometer (Agilent Technologies) in different ion modes (Supplementary Table 1).

#### Analysis

To evaluate which phospholipids could discriminate a patient with HCC from a patient without cancer, we compared the plasma values of each species for each defined phospholipid category. We chose the targeted HPLC-MS/MS approach with the multiple reaction monitoring (MRM) method to quantify specific species of these phospholipids in the plasma of the 90 cirrhotic patients. We decided to compare absolute quantification of controls and cases for future considerations in using reference values as a prognosis threshold. Concentrations of each phospholipid were determined from the ratio of the peak area of a given molecule to the peak area of the appropriate internal standard. Concentrations were calculated according to the quantity of internal standard and adjusted with the molecular weight of the corresponding species. Concentrations were expressed in micromoles per liter (μmol/L) of plasma (the plasma samples were analysed in triplicate). Isotopic overlap of lipid species and data analyses for all lipid classes were corrected using MassHunter Software (Agilent). Lipid species were annotated according to the recently published proposal for shorthand notation of lipid structures that are derived from mass spectrometry [39]. In cases where fatty acid composition was not speculated, the annotation represents the total number of carbons and double bonds.

### Statistical analysis

Percentages were used to describe categorical data, and medians and interquartile range to describe continuous data. Patient characteristics were Dcompared according to HCC status using the McNemar test (for categorical data) and the nonparametric Wilcoxon test (for continuous data) for paired samples. The association between the plasma concentration of phospholipid at inclusion and survival within 1 and 3 years in cases were assessed with Cox proportional hazards models, which estimated hazard ratios (HR) and 95% confidence intervals (CI). A p-value below 0.05 was considered significant. The statistical analyses were done using SAS version 9.4 software (SAS Institute Inc., Cary, NC, USA).

## SUPPLEMENTARY MATERIALS TABLES





## Abbreviations

ACLY: ATP citrate lyase
AFP: alpha fetoprotein
ATP: adenosine triphosp hate
BMI: body mass index
Cer: ceramide
CKα: choline kinase alpha
DHCer: dihydroceramide
FASN: fatty acid synthase
GGT: gammaglutamyl-transferases
HCC: hepatocellular carcinoma
HDL: high-density lipoprotein
HPLC-MSMS: high performance liquid chromatography coupled with tandem mass spectrometry
LDL: low-density lipoprotein
LPA: lysophosphatidic acid
LPC: lysophosphatidylcholine
LPCAT: lysophosphatidylcholine acyltransferase
LPE: lysophosphatidylethanolamine
LPL: lysophospholipid
MRM: multiple reaction monitoring
PC: phosphatidylcholine
pPC: plasmalogen-phosphatidylcholine
PE: phosphatidylethanolamine
PEMT: phosphatidylethanolamine N-methyltransferase
pPE: plasmalogen-phosphatidylethanolamine
PI: phosphatidylinositol
PL: phospholipid
PNPLA3: patatin-like phospholipase domain-containing protein 3
PS: phosphatidylserine
SM: sphingomyelin
UPLC/QTOF-MS: ultra performance liquid chromatography/quadrupole time-of-flight mass spectrometry
