# Supplementary material for: Phospholipid profiles and hepatocellular carcinoma risk and prognosis in cirrhotic patients
Source: Oncotarget. 2019 Mar 15;10(22):2161–72. doi: 10.18632/oncotarget.26738 (PMC6481329; doi:10.18632/oncotarget.26738)
Supplement: Supplementary file 1 [file oncotarget-10-2161-s001.pdf]

Phospholipid profiles and hepatocellular carcinoma risk and prognosis in cirrhotic patients

SUPPLEMENTARY MATERIALS

Supplementary Table 1: HPLC-MSMS parameters

Phospholipid	Standard	Quantity (µg)/ samples	Injection volume (µL)	LC column	Solvents	Ionisation mode	Spectrometer parameter
PC/pPC	di17:0 PC	10	4	Zorbax Eclipse Plus C18 2.1 × 100 mm; 1.8 µm	Mobile phase A: H2O/MeOH (60/40); 10 mM ammonium acetate;0.006% acetic acid Mobile phase B: Isopropanol/MeOH (90/10)	Positive ESI	MRM; precursor ion mode; product ion 184 m/z
LPC	17:0 LPC	1.2					
SM	17:0 SM	2					
PE/pPE	di17:0 PE	0.2	2				MRM; Neutral loss of 141 Da
LPE	14:0 LPE	0.2					
PI	di21:0 PC	0.2	2			Negative ESI	MRM; Neutral loss of 277 Da
Cer/DHCer	17:0 Cer	0.2	3	PL Proshell C8	Mobile phase A: H2O; Ammonium formate 1 mM; Formic acid 0.2% Mobile phase B: MeOH; Ammonium formate 1 mM; Formic acid 0.2%	Positive ESI	MRM; precursor ion mode; product ion 264 m/z

Supplementary Table 2: Phospholipid description.

See Supplementary File 1
